# Exploratory screening of wild-game tissues in Japan toward value-added utilization: estimated soluble protein/peptide yields and DPPH radical-scavenging and ACE-inhibitory activities after in vitro digestion

**DOI:** 10.1007/s44463-026-00118-0

**Published:** 2026-09-28

**Authors:** Hikaru Ogi, Takehiko Terashima, Ryo Mannen, Naoko Otsuki, Yoko Ichikawa

**Affiliations:** 1 Graduate School of Integrated Pharmaceutical and Nutritional Sciences, University of Shizuoka, Shizuoka, Japan; 2 Faculty of Health Promotional Sciences, Tokoha University, Hamamatsu, Japan; 3 Faculty of Nutritional Science, Sagami Women’s University, Sagamihara, Japan

**Keywords:** Game meat, Edible by-products, DPPH radical-scavenging activity, ACE inhibition

## Abstract

This exploratory study aimed to compare estimated soluble protein/peptide yields and 2,2-diphenyl-1-picrylhydrazyl (DPPH) radical-scavenging and angiotensin-converting enzyme (ACE)-inhibitory activities after in vitro digestion of eight tissues from wild-caught sika deer and wild boar and corresponding cattle and pig tissues, with relevance to value-added utilization in Japan. Eight tissues were obtained from sika deer and wild boar (n = 5 each) and commercially sourced cattle and pigs (n = 3 per tissue per species). Heated samples underwent gastric and intestinal digestion. DPPH and ACE activities were expressed as IC₅₀ values and analyzed using Kruskal–Wallis tests followed by Dunn’s tests with Holm adjustment. Estimated soluble protein/peptide yields differed among species groups in six tissues. Wild boar had higher yields in the stomach than sika deer, in the liver than sika deer and cattle, and in the round than pigs, but a lower yield in the small intestine than pigs. Sika deer spleen had a lower yield than wild boar and cattle spleens. DPPH IC₅₀ values were lower in wild boar liver than cattle liver and in wild boar loin than sika deer and cattle loin. ACE IC₅₀ was lower in sika deer spleen than wild boar spleen. Differences were tissue dependent, and estimated soluble protein/peptide yield did not consistently correspond to either activity. Wild boar liver and loin and sika deer spleen may warrant further characterization as candidates for value-added utilization in Japan.

## Introduction

In Japan, wild sika deer (*Cervus nippon*) and wild boar (*Sus scrofa*) have expanded their habitat ranges and continue to cause serious damage to agriculture, forestry, natural ecosystems, and living environments (Ministry of the Environment, Japan 2024; Ministry of Agriculture, Forestry and Fisheries, Japan 2024). To address these problems, population management through nuisance control and management culling has been implemented in many regions, and large numbers of sika deer and wild boar continue to be captured (Ministry of the Environment, Japan 2025). Game meat is considered an alternative source of animal protein (Morais et al., 2022), and expanded use may contribute to more sustainable food systems (Needham et al., 2023).

Commercial deer farming is established in several countries outside Japan (Kuba et al., 2015; Kudrnáčová et al., 2018). Sika deer themselves are also farmed for venison production, including in Germany and China (Rehbein & Visser, 2026; Peng et al., 2024). Thus, the production and utilization context of sika deer differs among regions. In Japan, however, only 3,059 captive deer were reported as of February 1, 2025 (Ministry of Agriculture, Forestry and Fisheries, Japan 2025a). In contrast, approximately 738,700 wild sika deer were captured in fiscal year 2024 (Ministry of the Environment, Japan 2025). Together with wild boars, the total number of captured animals reached approximately 1.38 million, of which only 167,760 (approximately 12.1%) were processed at wild-game meat processing facilities (Ministry of Agriculture, Forestry and Fisheries, Japan 2025a). Taken together, these national statistics suggest that game-meat utilization of deer and wild boar in Japan is based predominantly on wild-caught animals rather than on farmed production. Accordingly, the underutilization addressed in the present study refers specifically to wild-caught deer and wild boar resources in Japan, particularly tissues that are not routinely utilized for food (Ministry of Agriculture, Forestry and Fisheries, Japan 2025b; Ministry of Agriculture, Forestry and Fisheries, Japan 2026). Visceral tissues derived from domesticated livestock, such as cattle and pigs, have traditionally been consumed as food in many countries. These tissues are recognized as nutritious food materials rich in protein, essential amino acids, vitamins, and minerals (Toldrá et al., 2012). In addition, hydrolysates derived from livestock viscera have been reported to exhibit biological activities, including ACE-inhibitory and antioxidant activities (Inoue et al., 2013; Juknienė et al., 2023; Maheswarappa et al., 2023). Therefore, visceral tissues have attracted attention not only as by-products but also as potential functional food materials.

Research has increasingly examined the biological activities of hydrolysates derived from livestock viscera. In contrast, information on the biological activities of visceral tissues from wild animals, including sika deer and wild boar, remains limited. Few studies have compared the ACE-inhibitory and antioxidant activities of in vitro digests of wild-game visceral tissues with those of corresponding livestock tissues. The use of wild-game viscera for human consumption in Japan involves several challenges, including hygienic management at processing facilities, odors characteristic of wild animals, and limited consumer acceptance. Nevertheless, evaluating whether these tissues exhibit biological activities is important for assessing their potential value. Such fundamental knowledge may also provide a starting point for future research toward more comprehensive and sustainable use of wild-game resources.

This exploratory study evaluated estimated soluble protein/peptide yield, ACE-inhibitory activity, and DPPH radical-scavenging activity in in vitro digests of eight tissues obtained from wild-caught sika deer and wild boar. Corresponding commercially purchased cattle and pig tissues were evaluated for comparison. This study was not intended to establish the functionality of tissue digests from sika deer and wild boar, but rather to provide preliminary and fundamental information for the value-added utilization of underutilized wild-game resources in Japan. By applying a common heating, digestion, and analytical framework across eight tissues and four species groups, the study further aimed to characterize tissue-specific patterns in estimated soluble protein/peptide yield and two in vitro biological activities and to identify tissues warranting subsequent activity-guided characterization.

## Materials and methods

Between November 2024 and March 2025, samples were obtained from five female sika deer and five female wild boars, each estimated to be 3 years of age, captured in western Shizuoka Prefecture, Japan, and processed at a facility licensed for meat processing. Independently of this study, these animals had been captured using snare traps by licensed hunters as part of legally authorized population-management and commercial game-meat utilization activities. The authors informed the processing facility of the eligibility criteria for sex and estimated age but were not involved in the capture, transport, slaughter, or processing of the animals. Sampling was requested from animals brought to the processing facility for routine commercial use that met the specified sex and estimated-age criteria. The captured animals were transported alive to the processing facility and processed according to the facility’s routine procedures; the viscera were removed within 15 min after slaughter, and carcass processing was completed within 1 h. Heart, stomach, liver, spleen, small intestine, large intestine, loin, and round samples were then collected from each species (*n* = 5 per tissue). All samples were vacuum-packaged, rapidly frozen at -60 °C for 3 h, and stored at -30 °C. Corresponding cattle and pig tissues were purchased from a meat supplier in Shizuoka City, Japan, with each tissue obtained from three individual animals of each species (*n* = 3 per tissue per species). Before analysis, all samples were freeze-dried, pulverized using a food mill, and stored at -30 °C. The moisture content of each sample was calculated from the difference between the sample weights before and after freeze-drying. The pre-freeze-drying weight was treated as wet weight and the post-freeze-drying weight as dry weight, and moisture content was calculated using the following equation:

$$\:\mathrm{M}\mathrm{o}\mathrm{i}\mathrm{s}\mathrm{t}\mathrm{u}\mathrm{r}\mathrm{e}\:\mathrm{c}\mathrm{o}\mathrm{n}\mathrm{t}\mathrm{e}\mathrm{n}\mathrm{t}\:\left({\%}\right)=\left[\right(\mathrm{w}\mathrm{e}\mathrm{t}\:\mathrm{w}\mathrm{e}\mathrm{i}\mathrm{g}\mathrm{h}\mathrm{t}-\mathrm{d}\mathrm{r}\mathrm{y}\:\mathrm{w}\mathrm{e}\mathrm{i}\mathrm{g}\mathrm{h}\mathrm{t})\:/\mathrm{w}\mathrm{e}\mathrm{t}\:\mathrm{w}\mathrm{e}\mathrm{i}\mathrm{g}\mathrm{h}\mathrm{t}]\times 100$$

### Reagents

Pepsin from porcine gastric mucosa (P7012) was purchased from Sigma-Aldrich (St. Louis, MO, USA). Trypsin from porcine pancreas (207-19183) and pancreatin (163 − 00142) were obtained from FUJIFILM Wako Pure Chemical Corporation (Osaka, Japan). 2,2-Diphenyl-1-picrylhydrazyl (DPPH; D4313) was purchased from Tokyo Chemical Industry Co., Ltd. (Tokyo, Japan).

### In vitro simulated gastric and intestinal digestion

Samples were subjected to in vitro digestion using modified versions of previously reported methods (Takeda et al., 2015, 2020). First, to physically disrupt and homogenize the samples, 0.5 g of freeze-dried sample was weighed into a 15-mL tube, mixed with 4.5 mL of distilled water and 10 beads of Lysing Matrix S, Bulk (MP Biomedicals, Irvine, CA, USA), and disrupted using a FastPrep-24 Instrument (MP Biomedicals) at 6.0 m/s for 40 s. The samples were then heated at 70 °C for 30 min to provide a standardized heat treatment representing a cooked condition. After cooling, a 0.4 mL aliquot was collected from each heated sample and centrifuged at 2,000 × g for 5 min at 4 °C. The resulting supernatant was passed through a 0.45 μm syringe filter, and a 1 µL aliquot of the filtrate was applied to the measurement pedestal of a NanoDrop Lite Plus Microvolume Spectrophotometer (Thermo Fisher Scientific, Waltham, MA, USA). The apparent soluble protein concentration was estimated from the absorbance at 280 nm using the Protein A280 mode, with distilled water as the blank. This estimated concentration was used to calculate the amount of enzyme added to the remaining heated sample. For gastric digestion, the pH of the remaining heated sample was adjusted to 1.8 with HCl. Pepsin was added at a nominal enzyme-to-protein ratio of 1:1,000 (w/w), and the mixture was incubated at 37 °C for 2 h with gentle mixing. Pepsin was then inactivated by adjusting the pH to 6.8 with NaOH and heating at 100 °C for 10 min. After cooling to 40 °C, trypsin and pancreatin were each added at a nominal enzyme-to-protein ratio of 1:1,000 (w/w) to simulate intestinal digestion, and the mixture was incubated at 37 °C for an additional 2 h. The enzymes were inactivated by heating at 100 °C for 10 min. The digests were centrifuged at 2,000 × g for 5 min at 4 °C, and the supernatants were filtered through 0.45-µm syringe filters. The filtrates were collected for subsequent analyses.

### Estimated soluble protein/peptide yield

Estimated soluble protein/peptide concentration in each digest was calculated from the difference in absorbance at 215 and 225 nm according to the Waddell method (Waddell, 1956). The digest was diluted 400-fold with distilled water, and absorbance at 215 and 225 nm was measured using a UV–Vis spectrophotometer (UVmini-1240; Shimadzu Corporation, Kyoto, Japan). Estimated soluble protein/peptide concentration was calculated using the following equation:

$$\:\mathrm{E}\mathrm{s}\mathrm{t}\mathrm{i}\mathrm{m}\mathrm{a}\mathrm{t}\mathrm{e}\mathrm{d}\:\mathrm{s}\mathrm{o}\mathrm{l}\mathrm{u}\mathrm{b}\mathrm{l}\mathrm{e}\:\mathrm{p}\mathrm{r}\mathrm{o}\mathrm{t}\mathrm{e}\mathrm{i}\mathrm{n}/\mathrm{p}\mathrm{e}\mathrm{p}\mathrm{t}\mathrm{i}\mathrm{d}\mathrm{e}\:\mathrm{c}\mathrm{o}\mathrm{n}\mathrm{c}\mathrm{e}\mathrm{n}\mathrm{t}\mathrm{r}\mathrm{a}\mathrm{t}\mathrm{i}\mathrm{o}\mathrm{n}\:(\mathrm{m}\mathrm{g}/\mathrm{m}\mathrm{L})=\left({\mathrm{A}}_{215}-{\mathrm{A}}_{225}\right)\times 0.144$$

where A215 and A225 are the absorbance values at 215 and 225 nm, respectively.

The estimated soluble protein/peptide yield was converted to the equivalent amount per gram of raw wet tissue and expressed as mg estimated soluble protein/peptide/g wet tissue. Conversion to raw wet-tissue weight was based on the moisture content calculated from the difference in sample weight before and after freeze-drying. To assess the possible contribution of the digestive enzymes to the Waddell measurement, a single enzyme-only blank (*n* = 1) containing the digestive enzymes but no tissue sample was subjected to the same digestion and analytical procedure. The amounts of pepsin, trypsin, and pancreatin used for the enzyme-only blank were set to match those used in a representative tissue-digestion experiment. The estimated soluble protein/peptide concentration of the enzyme-only blank was determined using the Waddell method in the same manner as for the tissue digests.

### Measurement of DPPH radical-scavenging activity

DPPH radical-scavenging activity was measured using a modified version of the method described by Shimamura et al. (2014). The digests were used undiluted or diluted 4- or 10-fold with distilled water. An aliquot of each sample dilution (20 µL) and 0.1 M Tris–HCl buffer (pH 7.4; 80 µL) were added to a 96-well plate. For the control reaction, 20 µL of ethanol was added instead of the sample. Subsequently, 100 µL of 200 µM DPPH solution was added to initiate the reaction. After incubation in the dark at room temperature (20–25 °C) for 30 min, absorbance at 520 nm was measured using a microplate reader (iMark™, Bio-Rad Laboratories, Hercules, CA, USA). DPPH radical-scavenging activity was calculated using the following equation:

$$\:\text{DPPH radical scavenging activity }\left({\%}\right)=\left(\frac{{\mathrm{A}}_{\mathrm{c}\mathrm{o}\mathrm{n}\mathrm{t}\mathrm{r}\mathrm{o}\mathrm{l}}-{\mathrm{A}}_{\mathrm{s}\mathrm{a}\mathrm{m}\mathrm{p}\mathrm{l}\mathrm{e}}}{{\mathrm{A}}_{\mathrm{c}\mathrm{o}\mathrm{n}\mathrm{t}\mathrm{r}\mathrm{o}\mathrm{l}}}\right)\times 100$$

where Acontrol is the absorbance of the control reaction at 520 nm and Asample is the absorbance of the sample reaction at 520 nm.

### ACE-inhibitory activity

ACE-inhibitory activity was measured using the ACE Kit-WST (Dojindo Molecular Technologies, Inc., Kumamoto, Japan). The digests were assayed either undiluted or after 4- or 10-fold dilution with distilled water according to the manufacturer’s protocol for colored samples. Absorbance at 450 nm was measured using the iMark™ microplate reader. ACE inhibition was calculated using the following equation:

$$\text{ACE inhibitory activity}\left({\%}\right)=\left[\frac{{\mathrm{A}}_{\text{blank 1}}-\left({\mathrm{A}}_{\mathrm{sample}}-{\mathrm{A}}_{\text{sample blank}}\right)}{{\mathrm{A}}_{\text{blank 1}}-{\mathrm{A}}_{\text{blank 2}}}\right]\times 100$$

where Ablank_1_ is the absorbance of the uninhibited reaction control, Ablank_2_ is the absorbance of the reagent blank, Asample is the absorbance of the sample reaction, and Asample blank is the background absorbance of the sample.

The IC₅₀ values for DPPH radical-scavenging and ACE-inhibitory activities were estimated using Microsoft Excel 2019 (Microsoft Corporation, Redmond, WA, USA). For each assay, the measured responses were plotted against the final estimated soluble protein/peptide concentrations in the reaction mixture. IC₅₀ values were estimated by linear interpolation between the two adjacent measured concentrations that bracketed 50% activity, one producing a response below 50% and the other producing a response of at least 50%. No nonlinear dose–response curve fitting was performed. The final estimated soluble protein/peptide concentration in the reaction mixture that produced 50% DPPH radical-scavenging activity or ACE inhibition was defined as the IC₅₀ (mg estimated soluble protein/peptide/mL). IC₅₀ values were estimated only when two adjacent measured concentrations bracketed 50%; extrapolation beyond the tested concentration range was not performed.

### Statistical analyses

All measurements of tissue samples were performed in technical triplicate for each sample, and the mean of the three technical replicates was used as the representative value for statistical analysis. Statistical analyses were performed using R version 4.6.1 (R Foundation for Statistical Computing, Vienna, Austria). Differences among the four animal species were analyzed separately for each tissue using the Kruskal–Wallis test. When the Kruskal–Wallis test was significant, Dunn’s multiple-comparison test was performed using the rstatix package, and the six pairwise comparisons among the four species within each tissue–outcome combination were treated as one multiple-comparison family and adjusted using the Holm method. Each tissue–outcome combination was analyzed as a separate exploratory endpoint. All tests were two-sided, and Holm-adjusted *p*-values < 0.05 were considered statistically significant. Data are presented as individual observations, medians, and interquartile ranges (Q1–Q3).

## Results

### Estimated soluble protein/peptide yield

**Fig. 1:**
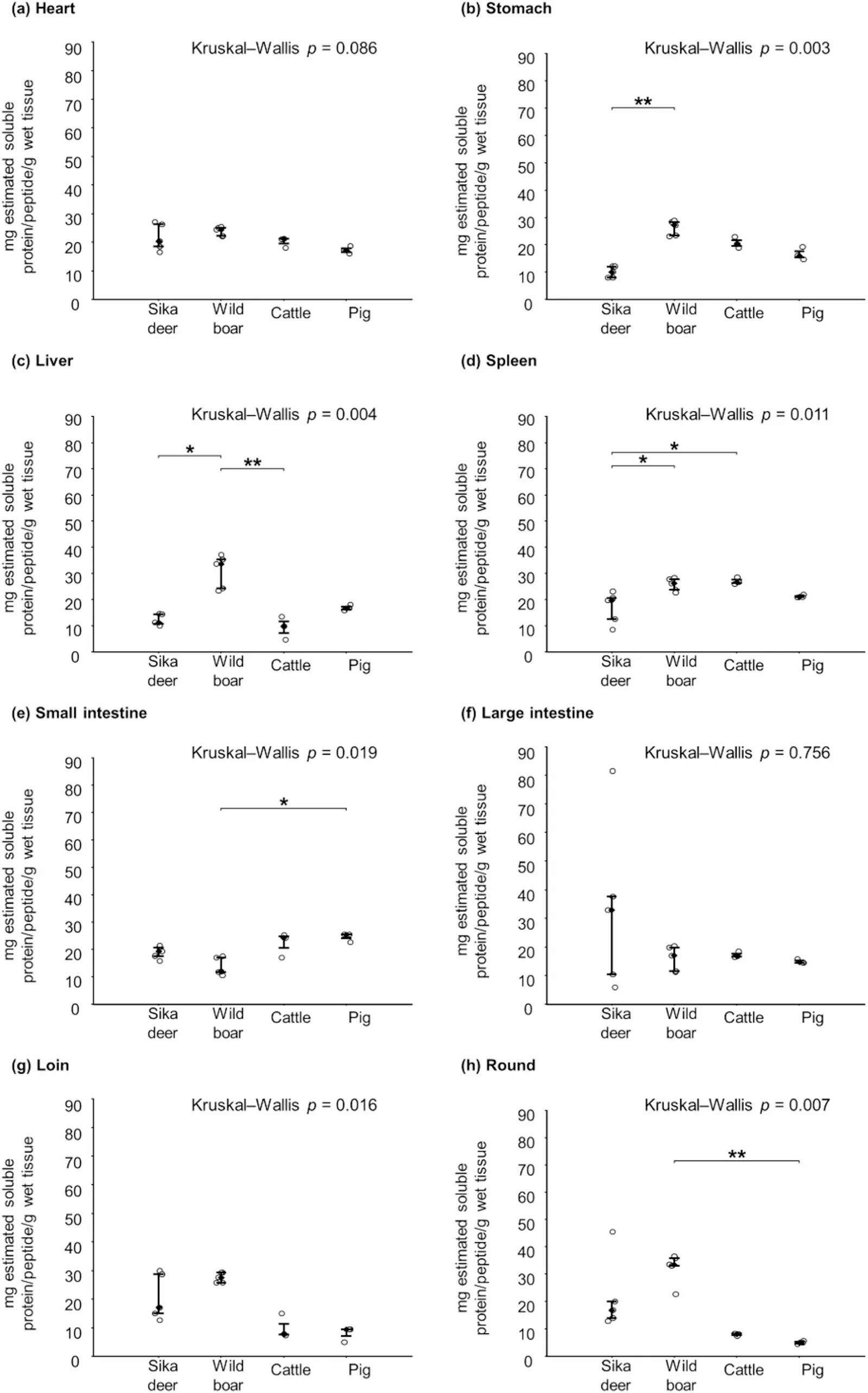
Estimated soluble protein/peptide yield after in vitro digestion of eight tissues from sika deer, wild boar, cattle, and pigs: (**a**) heart, (**b**) stomach, (**c**) liver, (**d**) spleen, (**e**) small intestine, (**f**) large intestine, (**g**) loin, and (**h**) round. Individual circles represent samples from individual animals (sika deer and wild boar, *n* = 5 individual animals each; cattle and pigs, *n* = 3 individual animals per tissue per species). Diamonds and error bars indicate the medians and interquartile ranges (Q1–Q3), respectively. Differences among species were analyzed using the Kruskal–Wallis test, followed by Dunn’s multiple-comparison test with Holm adjustment when the overall test was significant. Asterisks above brackets indicate significant pairwise differences based on Holm-adjusted *p*-values: *p < 0.05 and **p < 0.01

Estimated soluble protein/peptide yields were calculated per gram of wet tissue for each species and tissue. The Kruskal–Wallis test indicated significant differences among species in the stomach, liver, spleen, small intestine, loin, and round (Fig. 1). Following Dunn’s test with Holm adjustment, the estimated soluble protein/peptide yield in wild boar stomach was 27.14 [Q1–Q3: 23.46–28.26] mg estimated soluble protein/peptide/g wet tissue, which was significantly higher than that in sika deer stomach (10.05 [8.04–12.10] mg estimated soluble protein/peptide/g wet tissue; adjusted *p* = 0.0016) (Fig. 1b).

In the liver, the estimated soluble protein/peptide yield in wild boar was 33.59 [24.26–35.32] mg estimated soluble protein/peptide/g wet tissue, which was significantly higher than the corresponding yields in sika deer (11.17 [10.59–14.28] mg estimated soluble protein/peptide/g wet tissue; adjusted *p* = 0.0214) and cattle (9.76 [7.22–11.61] mg estimated soluble protein/peptide/g wet tissue; adjusted *p* = 0.0093) (Fig. 1c).

In the spleen, the estimated soluble protein/peptide yield in sika deer was 19.62 [12.55–20.65] mg estimated soluble protein/peptide/g wet tissue, which was significantly lower than the corresponding yields in wild boar (26.15 [23.77–27.83] mg estimated soluble protein/peptide/g wet tissue; adjusted *p* = 0.0394) and cattle (26.61 [26.30–27.59] mg estimated soluble protein/peptide/g wet tissue; adjusted *p* = 0.0367) (Fig. 1d).

In the small intestine, the estimated soluble protein/peptide yield in wild boar was 11.89 [11.72–16.98] mg estimated soluble protein/peptide/g wet tissue, which was significantly lower than that in pigs (25.34 [23.95–25.41] mg estimated soluble protein/peptide/g wet tissue; adjusted *p* = 0.0147) (Fig. 1e). Conversely, in the round, the estimated soluble protein/peptide yield in wild boar was 33.52 [33.12–35.92] mg estimated soluble protein/peptide/g wet tissue, which was significantly higher than that in pigs (4.98 [4.69–5.31] mg estimated soluble protein/peptide/g wet tissue; adjusted *p* = 0.0093) (Fig. 1h).

Although the Kruskal–Wallis test indicated a significant difference among species in the loin (*p* = 0.0157), no pairwise comparison remained significant after Holm adjustment. No significant differences among species groups were detected in the heart or large intestine by the Kruskal–Wallis test. A single enzyme-only blank (*n* = 1) analyzed using the same procedure showed an estimated soluble protein/peptide concentration of 0.059 mg/mL.

### DPPH radical-scavenging activity

**Fig. 2:**
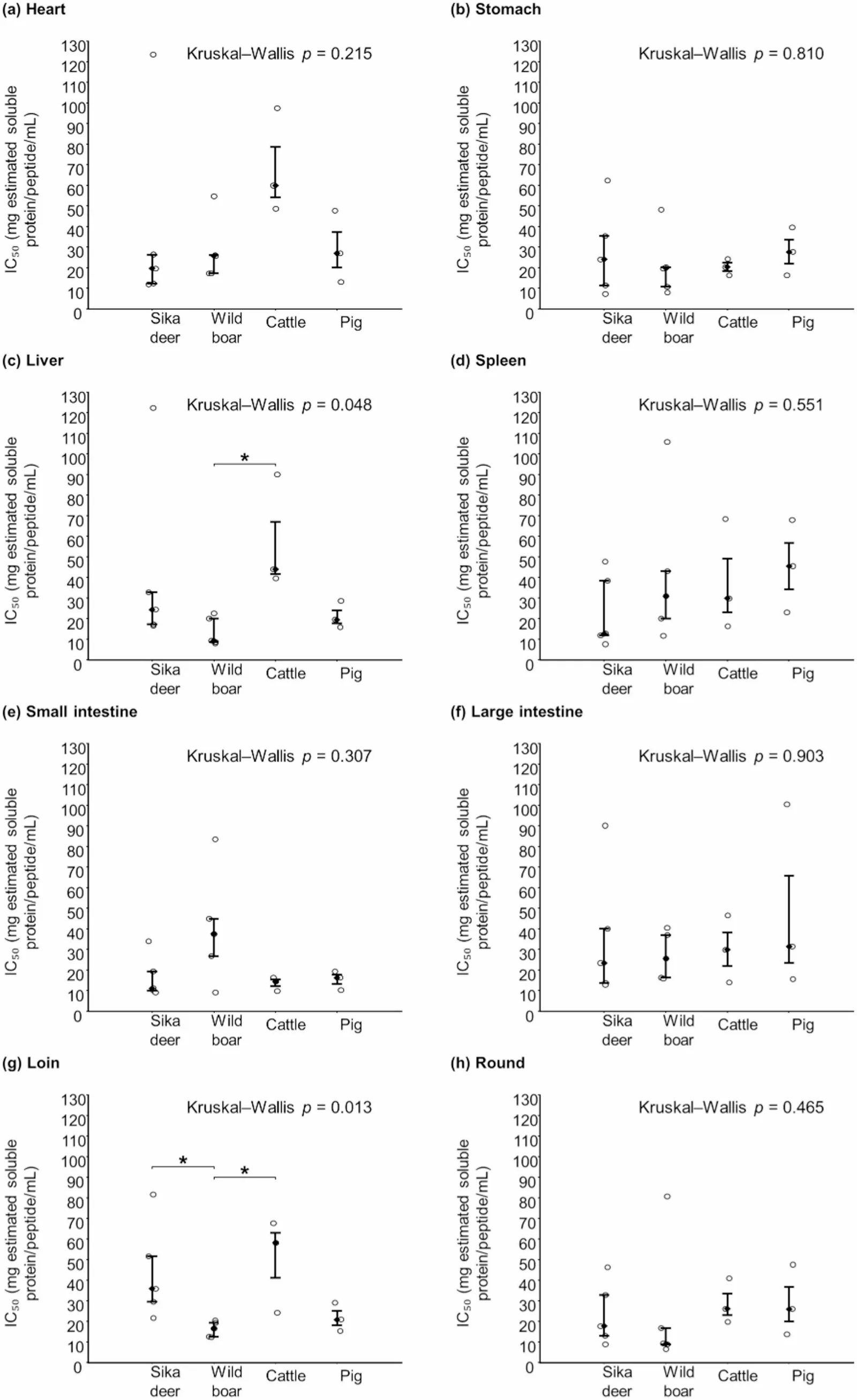
IC_50_ values for DPPH radical-scavenging activity after in vitro digestion of eight tissues from sika deer, wild boar, cattle, and pigs: (**a**) heart, (**b**) stomach, (**c**) liver, (**d**) spleen, (**e**) small intestine, (**f**) large intestine, (**g**) loin, and (**h**) round. Individual circles represent samples from individual animals (sika deer and wild boar, *n* = 5 individual animals each; cattle and pigs, *n* = 3 individual animals per tissue per species). Diamonds and error bars indicate the medians and interquartile ranges (Q1–Q3), respectively. Differences among species were analyzed using the Kruskal–Wallis test, followed by Dunn’s multiple-comparison test with Holm adjustment when the overall test was significant. Asterisks above brackets indicate significant pairwise differences based on Holm-adjusted *p*-values: *p < 0.05. Lower IC₅₀ values indicate that a lower estimated soluble protein/peptide concentration was required to achieve 50% DPPH radical scavenging under the present assay conditions

IC_50_ values were calculated for the DPPH radical-scavenging activity of the in vitro digests. The Kruskal–Wallis test indicated significant differences among species groups in the DPPH radical-scavenging IC_50_ values of the liver and loin (Fig. 2). Following Dunn’s test with Holm adjustment, the IC_50_ value of wild boar liver was 9.38 [8.79–20.09] mg estimated soluble protein/peptide/mL, which was significantly lower than that of cattle liver (44.02 [41.80–67.00] mg estimated soluble protein/peptide/mL; adjusted *p* = 0.0412) (Fig. 2c).

In the loin, the IC_50_ value in wild boar was 16.53 [12.45–19.32] mg estimated soluble protein/peptide/mL, which was significantly lower than those in sika deer (35.82 [29.39–51.54] mg estimated soluble protein/peptide/mL; adjusted *p* = 0.0317) and cattle (58.10 [41.11–62.86] mg estimated soluble protein/peptide/mL; adjusted *p* = 0.0456) (Fig. 2g).

No significant differences among species groups were detected in the heart, stomach, spleen, small intestine, large intestine, or round by the Kruskal–Wallis test.

### ACE-inhibitory activity

**Fig. 3:**
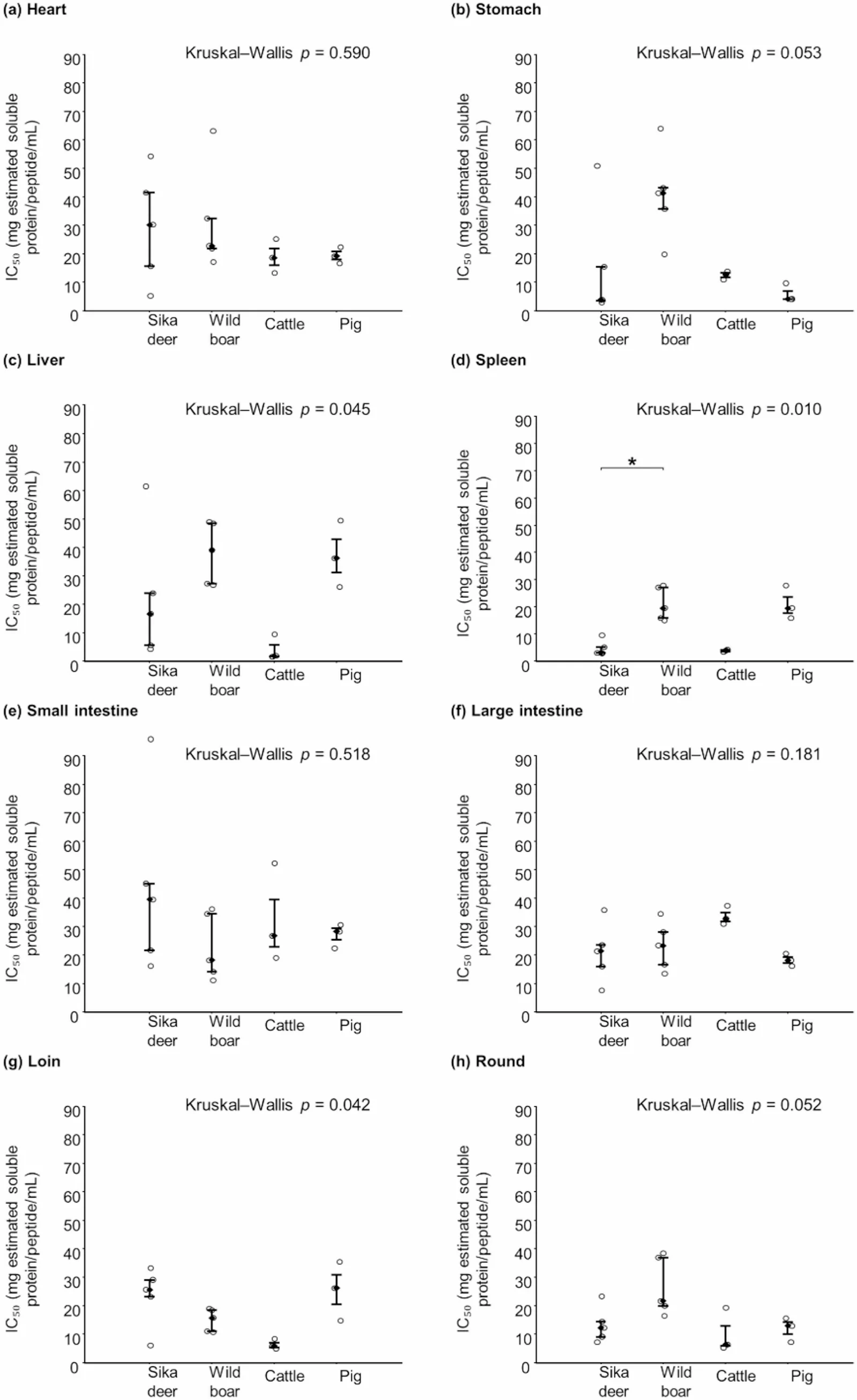
IC_50_ values for ACE-inhibitory activity after in vitro digestion of eight tissues from sika deer, wild boar, cattle, and pigs: (**a**) heart, (**b**) stomach, (**c**) liver, (**d**) spleen, (**e**) small intestine, (**f**) large intestine, (**g**) loin, and (**h**) round. Individual circles represent samples from individual animals (sika deer and wild boar, *n* = 5 individual animals each; cattle and pigs, *n* = 3 individual animals per tissue per species). Diamonds and error bars indicate the medians and interquartile ranges (Q1–Q3), respectively. Differences among species were analyzed using the Kruskal–Wallis test, followed by Dunn’s multiple-comparison test with Holm adjustment when the overall test was significant. Asterisks above brackets indicate significant pairwise differences based on Holm-adjusted *p*-values: *p < 0.05. Lower IC_50_ values indicate greater ACE-inhibitory activity

IC_50_ values were calculated for the ACE-inhibitory activity of the in vitro digests. The Kruskal–Wallis test indicated significant differences among species groups in the ACE-inhibitory IC_50_ values of the liver, spleen, and loin (Fig. 3). Following Dunn’s test with Holm adjustment, the IC_50_ value of sika deer spleen was 3.10 [3.08–5.16] mg estimated soluble protein/peptide/mL, which was significantly lower than that of wild boar spleen (19.48 [15.87–27.12] mg estimated soluble protein/peptide/mL; adjusted *p* = 0.0421) (Fig. 3d). Thus, under the present assay conditions, the sika deer spleen digest exhibited greater ACE-inhibitory activity than the wild boar spleen digest.

Although the Kruskal–Wallis test indicated significant differences among species groups in the liver and loin, no pairwise comparison remained significant after Holm adjustment. No significant differences among species groups were detected in the heart, stomach, small intestine, large intestine, or round by the Kruskal–Wallis test.

## Discussion

This study compared estimated soluble protein/peptide yields, DPPH radical-scavenging activity, and ACE-inhibitory activity after in vitro digestion of eight tissues from sika deer, wild boar, cattle, and pigs. Estimated soluble protein/peptide yields differed among species groups in several tissues, whereas significant pairwise differences in DPPH radical-scavenging and ACE-inhibitory activities were limited to a small number of comparisons. The biological activities of meat-derived peptides are reportedly influenced not only by the total amount of peptides produced but also by their source proteins, amino acid sequences, molecular weights, hydrophobicity, and charge (Lafarga & Hayes, 2014; Ryan et al., 2011; Jeong et al., 2024). Therefore, the present findings suggest that estimated soluble protein/peptide yield did not necessarily correspond to the measured biological activities.

The estimated soluble protein/peptide yield was higher in wild boar stomach than in sika deer stomach, higher in wild boar liver than in sika deer and cattle liver, and higher in wild boar round than in pig round. Conversely, the yield was lower in wild boar small intestine than in pig small intestine and lower in sika deer spleen than in wild boar and cattle spleen. Thus, the direction of differences among species groups was not consistent across tissues. Meat and meat by-products contain diverse proteins, and the generation and characteristics of bioactive peptides can vary depending on the protein source and hydrolysis or digestion conditions (Lafarga & Hayes, 2014; Ryan et al., 2011; Jeong et al., 2024). Therefore, the tissue-specific differences in estimated soluble protein/peptide yield observed in this study may have been related to differences in protein composition, tissue structure, and digestibility among the evaluated species groups. However, because tissue protein composition and post-digestion peptide profiles were not measured, the factors underlying these differences could not be identified.

For DPPH radical-scavenging activity, the IC_50_ value was lower in wild boar liver than in cattle liver and lower in wild boar loin than in sika deer and cattle loin. Meat proteins have been reported to generate antioxidant peptides through digestion or enzymatic hydrolysis (Lafarga & Hayes, 2014, Ryan et al., 2011; Jeong et al., 2024; Saiga et al., 2003). Antioxidant peptides have been obtained from porcine myofibrillar proteins following protease treatment (Saiga et al., 2003). In venison, heating and in vitro digestion alter DPPH radical-scavenging activity, and DPPH radical-scavenging peptides have been isolated from the resulting digests (Takeda et al., 2023).

Wild boar liver showed both a higher estimated soluble protein/peptide yield and a lower DPPH IC₅₀ than cattle liver. However, because the IC₅₀ was expressed on the basis of estimated soluble protein/peptide concentration, the lower IC₅₀ cannot be attributed simply to the higher yield and may instead reflect qualitative differences in the digests, including peptide composition and other water-soluble components. In contrast, although the Kruskal–Wallis test for estimated soluble protein/peptide yield in the loin was significant, pairwise comparisons between wild boar and sika deer or cattle were not significant after Holm adjustment. Nevertheless, a lower DPPH IC₅₀ was observed in wild boar under the present assay conditions. Therefore, the difference in loin DPPH IC₅₀ cannot be explained solely by the total estimated soluble protein/peptide yield and may instead be related to qualitative differences in the digests, including the types and sequences of digestion-derived peptides, free amino acids, and other water-soluble low-molecular-weight components.

The antioxidant activities of peptides are thought to be influenced by their molecular weights and by the types and positions of hydrophobic amino acids, such as leucine, isoleucine, and valine; aromatic amino acids, such as tryptophan, tyrosine, and phenylalanine; and histidine (Ryan et al., 2011; Jeong et al., 2024; Saiga et al., 2003). These amino acid residues may participate in electron or hydrogen donation, radical interactions, and metal-ion chelation. Therefore, peptides with sequences favorable for interaction with DPPH radicals may have been generated in wild boar liver and loin. However, because the amino acid sequences and molecular-weight distributions of the peptides were not determined, this interpretation remains speculative.

For ACE-inhibitory activity, the IC_50_ value was lower in sika deer spleen than in wild boar spleen; under the present assay conditions, the sika deer spleen digest therefore exhibited greater ACE-inhibitory activity. Although the Kruskal–Wallis test indicated differences among species groups in the liver and loin, no pairwise comparison remained significant after Holm adjustment. Therefore, no specific pairwise difference in ACE-inhibitory activity among the species groups can be concluded for these tissues.

Meat proteins have been reported to generate ACE-inhibitory peptides during digestion (Takeda et al., 2020; Escudero et al., 2010, 2012; Mora et al., 2018). The ACE-inhibitory peptides KAPVA and PTPVP have been identified in in vitro pork digests (Escudero et al., 2010), and antihypertensive effects of some of these peptides have been reported in spontaneously hypertensive rats (Escudero et al., 2012). ACE-inhibitory activity has also been measured in sika deer and wild boar meat digests, and ACE-inhibitory peptides have been identified from sika deer meat (Takeda et al., 2020). These reports support the possibility that digestion of wild-game meat, as well as livestock meat, can generate ACE-inhibitory peptides.

Although the estimated soluble protein/peptide yield in sika deer spleen was lower than that in wild boar spleen, ACE-inhibitory activity was greater in sika deer. This finding further indicates that ACE-inhibitory activity cannot be explained solely by the estimated soluble protein/peptide yield. ACE-inhibitory activity is strongly influenced by peptide molecular weight, hydrophobicity, charge, and amino acid sequences near the C-terminus (Jeong et al., 2024; Escudero et al., 2010; Mora et al., 2018). Thus, although the estimated soluble protein/peptide yield was lower in sika deer spleen, peptides with structures favorable for ACE binding may have been relatively abundant. However, because individual bioactive peptides were not identified, the present study does not directly demonstrate their presence.

The animal species and tissues showing significant differences were not the same for DPPH radical-scavenging and ACE-inhibitory activities. Significant differences involving wild boar liver and loin were observed for DPPH radical-scavenging activity, whereas the significant difference in ACE-inhibitory activity involved sika deer and wild boar spleens. This discrepancy may reflect differences between peptide structures favorable for reaction with DPPH radicals and those favorable for binding to the active site of ACE. Therefore, a lower DPPH IC₅₀ in a digest does not necessarily indicate greater ACE-inhibitory activity, and the two activities should be evaluated separately.

An important strength of this study is its tissue-resolved, multi-endpoint comparative design. The academic contribution of this approach lies in systematically characterizing tissue-specific activity patterns under a common experimental framework. Eight tissues from two wild species and two livestock species were processed using a common heating and in vitro digestion procedure and evaluated within the same analytical framework. This approach enabled comparison of estimated soluble protein/peptide yield with two distinct biological activities and revealed that the activity patterns were tissue dependent and did not simply correspond to the amount of soluble protein/peptide material. The lack of a consistent correspondence between estimated soluble protein/peptide yield and the two activities also suggests that screening based solely on soluble material recovery may not identify tissues with distinctive in vitro activity profiles. Furthermore, wild boar liver and loin and sika deer spleen may represent candidate tissues for subsequent activity-guided fractionation and characterization of the components contributing to the observed activities. Thus, the present findings provide a systematic screening basis for future research on the value-added utilization of underused wild-game tissues.

Several limitations should be considered when interpreting the present findings. First, the study included five sika deer and five wild boars, whereas only three individual cattle and three individual pigs were evaluated per tissue, resulting in small and unequal sample sizes for each tissue comparison. Statistical power was therefore limited, and the effects of biological variability may not have been fully evaluated. In particular, a non-significant comparison indicates only that no detectable difference was identified under the conditions of this study and does not demonstrate equivalence among animal species. Moreover, when the overall test was significant but no pairwise comparison remained significant after Holm adjustment, no specific difference between two groups can be concluded. Multiplicity adjustment was applied to pairwise comparisons within each tissue–outcome combination but not across the full set of tissues and outcomes; therefore, the findings should be interpreted as exploratory and hypothesis-generating. In addition to animal species, the groups differed in wild-caught versus domesticated status, sex and age information, diet and living or rearing environments, sample provenance, and postmortem processing conditions. Consequently, the observed differences cannot be attributed to animal species alone. The findings should therefore be interpreted as an exploratory comparison of samples obtained under different conditions and evaluated using a common in vitro digestion procedure. Because enzyme addition was based on an A280-derived estimate of apparent soluble protein rather than an independent measurement of total tissue protein, differences in the effective enzyme-to-substrate ratio among tissues cannot be completely excluded. In addition, because the samples were heated at 70 °C before digestion, the present findings apply to the standardized cooked-condition model used in this study and should not be generalized to the digestion of raw tissues. Larger studies with prespecified designs and more balanced sample sizes are therefore required to validate these exploratory findings.

Second, an enzyme-only digestion blank was not measured concurrently with the original tissue digests. A single enzyme-only blank (*n* = 1) was subsequently subjected to the same digestion and analytical procedure, and its estimated soluble protein/peptide concentration was 0.059 mg/mL. Because this assessment was based on a single additional blank experiment and was not performed concurrently with the original tissue digests, the blank value was not retrospectively subtracted from the reported data. In addition, the Waddell method used in this study estimates concentration from absorbance in the short-wavelength UV region, and interference from pigments, nucleotide-related compounds, free amino acids, and other UV-absorbing components in visceral digests cannot be completely excluded. The reported values should therefore be interpreted as estimated soluble protein/peptide concentrations rather than as peptide-specific measurements. Because the estimated soluble protein/peptide concentration was also used as the concentration scale for IC₅₀ calculation, uncertainty in the Waddell-derived estimates may have affected the absolute IC₅₀ values.

Third, the DPPH assay is an in vitro screening method based on a stable synthetic radical, and its results are influenced by factors such as reaction time, solvent, and pH (Xie & Schaich, 2014). Therefore, the DPPH radical-scavenging activities observed in this study cannot be directly extrapolated to antioxidant effects in vivo. Moreover, the digests were diluted with distilled water, whereas ethanol was used in the control reaction; therefore, the solvent compositions of the sample and control reactions were not completely matched. In addition, sample-specific absorbance at 520 nm was not corrected using sample blanks without DPPH. Particularly for colored visceral digests, effects of differences in solvent composition and matrix-derived absorbance on the measurements cannot be excluded.

Calculated ACE-inhibition values exceeding 100% were obtained for some undiluted samples. However, inhibition decreased with sample dilution, and IC₅₀ values were calculated only from measured concentrations that bracketed 50% inhibition without extrapolation beyond the tested range. Similarly, ACE-inhibitory activity indicates that the digests inhibited ACE under in vitro conditions and does not directly demonstrate an antihypertensive effect after ingestion. Furthermore, because standard antioxidants such as Trolox and standard ACE inhibitors such as captopril were not measured concurrently, the absolute potencies of the obtained IC_50_ values cannot be compared with those of established reference compounds.

For an ACE-inhibitory peptide to exert an effect in vivo, it must remain stable in the gastrointestinal tract, be absorbed through the intestinal epithelium, remain stable in the circulation, and reach its site of action (Escudero et al., 2012; Mora et al., 2018). Future studies should include optimization of heating, digestion, and extraction conditions, molecular-weight fractionation using ultrafiltration or chromatography, identification of peptide sequences by LC–MS/MS, activity confirmation using synthetic peptides, and evaluation of physiological efficacy in cell-based and animal studies.

Overall, estimated soluble protein/peptide yields, DPPH radical-scavenging activities, and ACE-inhibitory activities measured after in vitro digestion varied among species–tissue groups. In particular, wild boar liver and loin and sika deer spleen may warrant further investigation of peptides and other components contributing to the observed activities.

## Conclusion

This exploratory study showed that the measured outcomes varied among species–tissue groups under the present experimental conditions. The DPPH IC₅₀ was lower in wild boar liver than in cattle liver and lower in wild boar loin than in sika deer and cattle loin. Because sample-specific absorbance at 520 nm was not corrected, matrix-derived absorbance, particularly in colored tissue digests, may have influenced the DPPH measurements; therefore, the DPPH findings should be interpreted cautiously. The ACE IC₅₀ was also lower in sika deer spleen than in wild boar spleen. However, estimated soluble protein/peptide yield did not consistently correspond to either activity, suggesting that qualitative characteristics of the digests, including peptide composition and other water-soluble low-molecular-weight components, may have contributed to the observed activities. Wild boar liver and loin and sika deer spleen may therefore warrant further investigation of peptides and other components contributing to the observed assay responses. Further studies are required to isolate active fractions, identify peptides and other active components, and evaluate their biological relevance in appropriate physiological models.

## Data Availability

No datasets were generated or analysed during the current study.
